# Comparative evaluation of 4 commercial modified‐live porcine reproductive and respiratory syndrome virus (PRRSV) vaccines against heterologous dual Korean PRRSV‐1 and PRRSV‐2 challenge

**DOI:** 10.1002/vms3.282

**Published:** 2020-05-21

**Authors:** Taehwan Oh, Su‐Jin Park, Hyejean Cho, Siyeon Yang, Hee Jin Ham, Chanhee Chae

**Affiliations:** ^1^ College of Veterinary Medicine Department of Veterinary Pathology Seoul National University Gwanak‐gu Seoul Republic of Korea; ^2^ College of Liberal Arts Anyang University Anyang‐si Republic of Korea

**Keywords:** co‐infection, modified‐live virus vaccine, porcine reproductive and respiratory syndrome, vaccination

## Abstract

**Background:**

Four commercial porcine reproductive and respiratory syndrome virus (PRRSV) modified‐live vaccines (MLV) was compared to protect growing pigs against dual challenge of PRRSV‐1 and PRRSV‐2.

**Methods:**

Two of the vaccines were based on PRRSV‐1, and two on PRRSV‐2. A total of 72 PRRSV‐naïve pigs were divided into six groups (12 pigs/group).

**Results:**

Two PRRSV‐1 MLV‐vaccinated and two PRRSV‐2 MLV‐vaccinated groups reduced significantly (*p* < .05) genomic copies of PRRSV‐1 in their sera compared to the unvaccinated challenged group. Two PRRSV‐2 MLV‐vaccinated groups reduced significantly (*p* < .05) fewer genomic copies of PRRSV‐2 in their sera whereas two PRRSV‐1 MLV‐vaccinated groups were unable to reduce genomic copies of PRRSV‐2 compared to unvaccinated challenged groups. Two PRRSV‐1 MLV‐vaccinated groups induced a stronger PRRSV‐1 specific IFN‐γ‐SC response, while two PRRSV‐2 MLV‐vaccinated groups induced a stronger PRRSV‐2 specific IFN‐γ‐SC response. Two PRRSV‐2 MLV‐vaccinated groups showed significantly (*p* < .05) lower mean macroscopic and microscopic lung lesion scores compared to two PRRSV‐1 MLV‐vaccinated groups.

**Conclusions:**

These data demonstrated that two PRRSV‐2 vaccines were efficacious and exhibited similar protection while, two PRRSV‐1 vaccines were largely ineffective against the dual challenge.

## Graphical abstract

Four commercial porcine reproductive and respiratory syndrome virus (PRRSV) modified‐live vaccines (MLV) was compared to protect growing pigs against dual challenge of PRRSV‐1 and PRRSV‐2. These study demonstrated that two PRRSV‐2 vaccines were efficacious and exhibited similar protection while, two PRRSV‐1 vaccines were largely ineffective against the dual challenge.

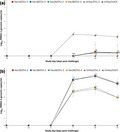

## 1 INTRODUCTION

Porcine reproductive and respiratory syndrome virus (PRRSV), which belongs to the family *Arteriviridae* and the order *Nidovirales* causes great economic losses to the swine industry worldwide (Snijder, Kikkert, & Fang, 2013). Infection with PRRSV is characterized by reproductive failure (abortions, premature farrowing and weak or stillborn animals) in gilts and sows, and respiratory disease in growing pigs. PRRSV can be divided into two distinct species, PRRSV‐1 (former genotype 1 from European‐like strain) and PRRSV‐2 (former genotype 2 from North American‐like strain). PRRSV‐2 was first discovered in Korea in 1994 and later PRRSV‐1 was isolated in 2005 (Kim et al., 2010; Kweon et al., 1994). Since then, co‐infection with both species within the same farm has become endemic (Choi, Lee, Park, Jeong, & Chae, 2015).

As co‐infection with both species becomes more prevalent, controlling co‐infections has rapidly become the main focus for swine producers. Theoretically, concurrent vaccination of pigs with both PRRSV‐1 and PRRSV‐2 vaccines has the potential to control co‐infection of pigs with both species. However, a recent study has suggested that concurrent vaccination of pigs with a PRRSV‐1 and a PRRSV‐2 modified‐live virus (MLV) vaccine may interfere with the efficacy of the PRRSV‐2 vaccine (Park et al., 2015). Therefore, control of both species with a single PRRSV vaccine is currently a major clinical focus. Currently, there are four commercially available PRRSV MLV vaccines in the Korean market, two based on PRRSV‐1 and two based on PRRSV‐2. A previous study showed that a PRRSV‐2 MLV vaccine was efficacious against dual PRRSV‐1 and PRRSV‐2 challenge (Choi et al., 2016). However, to date, no comparative study has been conducted to compare all four commercially available PRRSV‐MLV vaccines against heterologous dual PRRSV‐1 and PRRSV‐2 challenge under the same experimental conditions. Therefore, the objective of this study was to evaluate and compare the efficacy of all four commercial PRRSV MLV vaccines against respiratory disease of caused by heterologous dual PRRSV‐1 and PRRSV‐2 challenge in growing pigs based on clinical, virological, immunological and pathological analyses.

## 2 MATERIALS AND METHODS

### 2.1 Commercial vaccines

**Figure 1:**
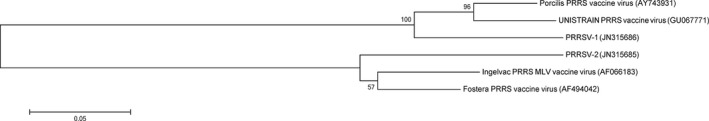
Phylogenetic analysis. Open reading frame 5 genome from the challenge PRRSV and the vaccine viruses. An unrooted neighbour‐joining tree was constructed from aligned nucleotide sequences

PRRSV‐1 (SNUVR090485, pan‐European subtype 1) and PRRSV‐2 (SNUVR090851, lineage 1) were used as inocula (Han et al., 2012, 2013). Open reading frame 5 (ORF5) sequence of the vaccine strains was compared with the challenge strains. The PRRSV‐1 (SNUVR090485) challenge strain shares a 87.9%, 88.1%, 61.1% and 61.1% identity with Porcilis PRRS, UNISTRAIN PRRS, Ingelvac PRRS MLV and Fostera PRRS, respectively. The PRRSV‐2 (SNUVR090851) challenge strain shares a 59.5%, 59.3%, 85.9% and 87.1% identity with Porcilis PRRS, UNISTRAIN PRRS, Ingelvac PRRS MLV and Fostera PRRS, respectively (Figure 1).

### 2.2 Experimental design

A total of 72 PRRSV‐naïve pigs from a PRRSV‐free farm were selected for this study. All pigs were confirmed negative for PRRSV by serology testing and by quantitative RT‐PCR on the day of vaccination. Pigs were divided into six groups (12 pigs/group) and assigned into six rooms using the random number generation function (Excel, Microsoft Corporation, Redmond, Washington, USA). Pigs in each of group were housed in same room (Table 1). This study is basically new dual challenge study and new pigs were actually infected for this current study.

**Table 1:** Experimental design and means (with standard deviation) of lung lesion score in pigs from various groups with 7 and 10 days post challenge (dpc)

Groups	PRRSV		dpc	Lung lesion score	
	Vaccination (28 days)	Challenge (63 days)		Macroscopic	Microscopic
Vac1A/Ch1−2	Porcilis PRRS	PRRSV−1 & −2	7	63.17±12.01^a^	3.25±0.42^a^
			14	46.67±10.33^a^	2.5±0.55^a^
Vac1B/Ch1−2	UNISTRAIN PRRS	PRRSV−1 & −2	7	64.17±10.21^a^	3.33±0.52^a^
			14	48.33±9.83^a^	2.67±0.82^a^
Vac2A/Ch1−2	Ingelvac PRRS MLV	PRRSV−1 & −2	7	32.5±7.58^b^	2.42±0.49^b^
			14	23.33±15.38^b^	1.42±0.49^b^
Vac2B/Ch1−2	Fostera PRRS	PRRSV−1 & −2	7	33.33±8.76^b^	2.33±0.52^b^
			14	24.17±11.58^b^	1.33±0.52^b^
UnVac/Ch1−2	None	PRRSV−1 & −2	7	63.33±10.33^a^	3.83±0.41^a^
			14	53.33±10.33^a^	3±0.63^a^
UnVac/UnCh	None	None	7	0.83±2.04^c^	0.17±0.41^c^
			14	0^c^	0.17±0.41^c^
					

Note

Different letters (a, b and c) at days post challenge indicate significant differences among groups.

At − 35 days post challenge (dpc, 28 days of age), pigs were injected intramuscularly on the right side of the neck with 2 ml of Porcilis PRRS (Vac1A/Ch1‐2 group, MSD Animal Health, Lot No. D353A07), UNISTRAIN PRRS (Vac1B/Ch1‐2 group, Hipra, Lot No. 61WK‐B), Ingelvac PRRS MLV (Vac2A/Ch1‐2, Boehringer Ingelheim Vetmedica, Lot No. 245‐659A) and Fostera PRRS (Vac2B/Ch1‐2 group, Zoetis, Lot No. A405013B) according to the manufacturer's instructions. The pigs in the UnVac/Ch1‐2 and UnVac/UnCh groups were administered an equal volume of phosphate buffered saline (PBS, 0.01M, pH 7.4).

At 0 dpc (63 days of age), the pigs in the Vac1A/Ch1‐2, Vac1B/Ch1‐2, Vac2A/Ch1‐2, Vac2B/Ch1‐2 and UnVac/Ch1‐2 groups were inoculated intranasally with 3 ml of PRRSV‐1 (10^5^ TCID_50_/mL of SNUVR090485, second passage in alveolar macrophages) and PRRSV‐2 (10^5^ TCID_50_/mL of SNUVR090851, second passage in alveolar macrophages) inoculum. Pigs in UnVac/UnCh were inoculated intranasally with 3 ml of PBS and served as the negative control group. Oral fluids were collected from all pigs prior to challenge (Prickett et al., 2008).

Upon challenge with PRRSV‐1 and PRRSV‐2, pigs in the Vac1A/Ch1‐2, Vac1B/Ch1‐2, Vac2A/Ch1‐2, Vac2B/Ch1‐2 and UnVac/Ch1‐2 groups were randomly assigned into five of six rooms using the random number generation function (Excel; Microsoft Corporation). Each room had 12 pens and pigs were housed individually in each pen. The pigs in the UnVac/UnCh group were randomly placed into 12 pens in the one remaining room. Following PRRSV challenge, the physical condition of each pig was monitored daily and rectal temperatures were recorded. From each group, six pigs were randomly selected using the random number generation function (Excel; Microsoft Corporation) at 7 and 14 dpc. Pigs were, sedated by an intravenous injection of sodium pentobarbital, euthanized by electrocution and necropsied (Beaver et al., 2001).

### 2.3 Clinical observation

Clinical respiratory observations were also recorded daily using scores ranging from 0 (normal) to 6 (severe dyspnoea and abdominal breathing) (Halbur et al., 1995). Observers were blinded to vaccination and challenge status. Rectal temperatures were recorded daily at the same time by same personnel.

### 2.4 Quantification of PRRSV RNA

RNA was extracted from serum samples to quantify PRRSV genomic cDNA copy numbers. PRRSV‐1 forward and reverse primers were 5'‐ TGGCCAGTCAGTCAATCAAC‐3' and 5'‐AATCGATTGCAA GCAGAGGGAA‐3', respectively. PRRSV‐2 forward and reverse primers were 5'‐TGGCCAGTCAGTCAATCAAC‐3' and 5'‐AATCGATTGCAAGCAGAGGGAA‐3', respectively (Halbur et al., 1995; Wasilk et al., 2004) Real‐time PCR for the PRRSV‐1 and PRRSV‐2 were performed to quantify PRRSV genomic cDNA copy (Halbur et al., 1995; Wasilk et al., 2004). Real‐time PCR for the vaccine viruses was also performed to quantify PRRSV genomic cDNA copy (Han, Seo, Park, & Chae, 2014; Kim et al., 2015; Park, Seo, Han, Kang, & Chae, 2014).

### 2.5 Serology

Blood samples were collected at −35, −21, 0, 7, 10 and 14 dpc. The samples were tested using a commercially available PRRSV enzyme‐linked immunosorbent assay (ELISA; HerdCheck PRRS X3 Ab test, IDEXX Laboratories Inc). Samples were considered positive if the sample‐to‐positive (S/P) ratio was ≥ 0.4, according to the manufacturer's instructions.

### 2.6 Enzyme‐linked immunospot assay

The number of PRRSV‐specific interferon‐γ secreting cells (IFN‐γ‐SC) was determined in vitro by stimulating peripheral blood mononuclear cells (PBMC) with the PRRSV‐1 and PRRSV‐2 challenge strains by enzyme‐linked immuospot (ELLISPOT) assay (Kim et al., 2015; Meier et al., 2003; Park et al., 2014).

### 2.7 Pathology

The severity of macroscopic lung lesions was scored to estimate the percentage of the lung affected by pneumonia. The scoring was done by two pathologists at the institution where this study was performed. For the entire lung, 100 points were assigned as follows; 10 points each to the right cranial lobe, right middle lobe, left cranial lobe, and left middle lobe, 27.5 points each to the right caudal lobe and left caudal lobe and 5 points to the accessory lobe (Halbur et al., 1995).

Microscopic lung lesions were also blindly assessed by the pathologists. Lesions were scored on a scale from 0 to 4:0 = no microscopic lesions; 1 = mild interstitial pneumonia; 2 = moderate multifocal interstitial pneumonia; 3 = moderate diffuse interstitial pneumonia; and 4 = severe interstitial pneumonia (Halbur et al., 1995).

### 2.8 Statistical analysis

Statistical analyses were performed using SPSS software (version 21; IBM, Armonk, New York). Prior to statistical analysis, RT‐PCR data were transformed to log_10_ values. Data were tested for normal distribution using the Shapiro‐Wilk test. One‐way analysis of variance (ANOVA) was used to examine whether there were statistically significant differences among the four groups, for each time point. When a test result from one‐way ANOVA showed a statistical significance, a post‐hoc test was conducted for a pairwise comparison with Tukey's adjustment. If the normality assumption was not met, the Kruskal‐Wallis test was performed. When the result form Kruskal‐Wallis test showed statistical significance, Mann‐Whitney test with Tukey's adjustment was performed to compare the differences among the groups. A value of *p* < .05 was considered to be significant.

## 3 RESULTS

### 3.1 Clinical observation

**Figure 2:**
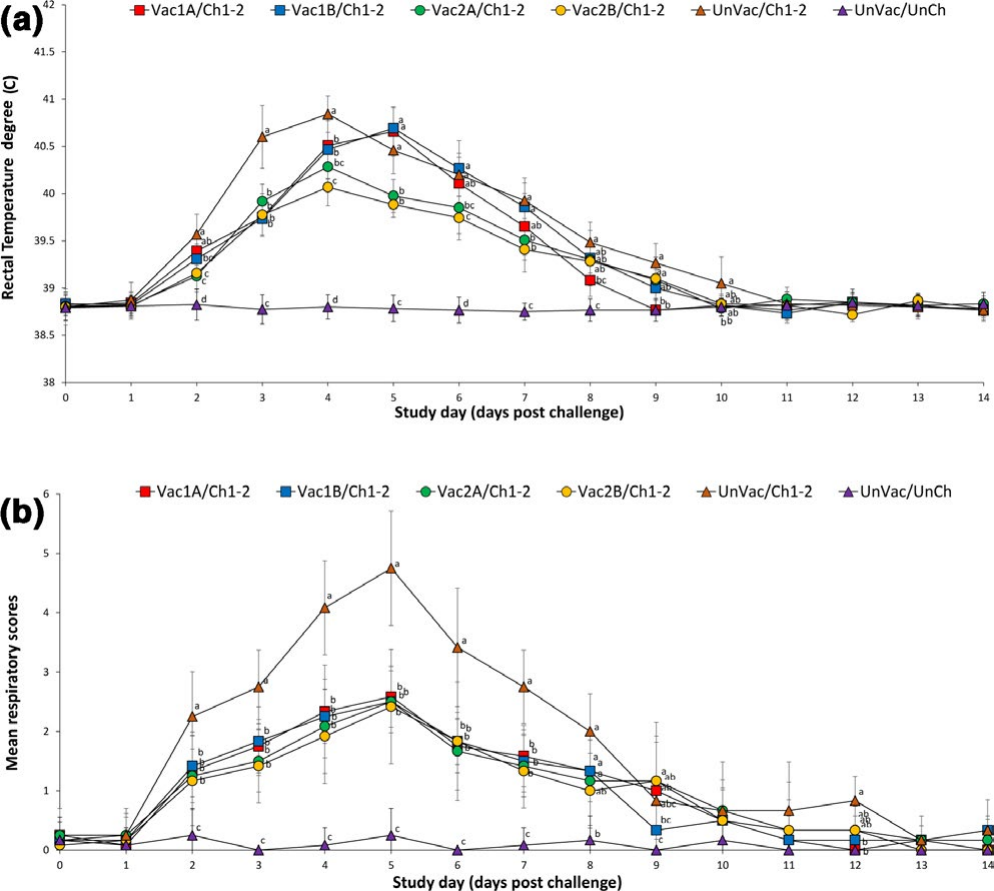
Mean (with standard deviation) rectal temperature (a) and mean (with standard deviation) respiratory scores (b) of pigs from six groups. Different letters (a, b and c) at days post challenge indicate significant differences among groups

There were no clinical signs observed in any of the groups after vaccination and before challenge. Following challenge, the mean rectal temperature was significantly lower (*p* < .05) in pigs from the Vac2A/Ch1‐2 and Vac2B/Ch1‐2 groups at 2 dpc compared to pigs from the Vac1A/Ch1‐2 and UnVac/Ch1‐2 groups. The mean rectal temperature was significantly (*p* < .05) lower in pigs from the Vac1B/Ch1‐2 at 2 dpc compared to pigs from the UnVac/Ch1‐2 group. The mean rectal temperature was significantly lower (*p* < .05) in pigs from the Vac1A/Ch1‐2, Vac1B/Ch1‐2, Vac2A/Ch1‐2 and Vac2B/Ch1‐2 groups at 3 and 4 dpc compared to pigs from the UnVac/Ch1‐2 group. The mean rectal temperature was significantly lower (*p* < .05) in pigs from the Vac2B/Ch1‐2 group at 4 dpc compared to pigs from the Vac1A/Ch1‐2 and Vac1B/Ch1‐2 groups. The mean rectal temperature was significantly lower (*p* < .05) in pigs from the Vac2A/Ch1‐2 and Vac2B/Ch1‐2 groups at 5 dpc compared to pigs from the Vac1A/Ch1‐2, Vac1B/Ch1‐2, and UnVac/Ch1‐2 groups. The mean rectal temperature was significantly lower (*p* < .05) in pigs from the Vac2B/Ch1‐2 at 6 dpc compared to pigs from the Vac1A/Ch1‐2, Vac1B/Ch1‐2 and UnVac/Ch1‐2 groups. The mean rectal temperature was significantly (*p* < .05) lower in pigs from the Vac2A/Ch1‐2 group at 6 dpc compared to pigs from the Vac1B/Ch1‐2 and UnVac/Ch1‐2 groups. The mean rectal temperature was significantly lower (*p* < .05) in pigs from the Vac2A/Ch1‐2 and Vac2B/Ch1‐2 groups at 7 dpc compared to pigs from the Vac1B/Ch1‐2 and UnVac/Ch1‐2 groups. The mean rectal temperature was significantly lower (*p* < .05) in pigs from the Vac1A/Ch1‐2 group at 8 dpc compared to pigs from UnVac/Ch1‐2 group. The mean rectal temperature was significantly lower (*p* < .05) in pigs from the Vac1A/Ch1‐2 group at 9 dpc compared to pigs from the Vac2A/Ch1‐2, Vac2B/Ch1‐2, and UnVac/Ch1‐2 groups. The mean rectal temperature was significantly lower (*p* < .05) in pigs from the Vac2A/Ch1‐2 group at 10 dpc compared to pigs from the UnVac/Ch1‐2 group (Figure 2a).

Pigs from the Vac1A/Ch1‐2, Vac1B/Ch1‐2, Vac2A/Ch1‐2 and Vac2B/Ch1‐2 groups had significantly lower (*p* < .05) respiratory sign scores between 2 and 7 dpc compared to pigs from the UnVac/Ch1‐2 group. Interestingly, pigs from the Vac1B/Ch1‐2 group at 9 dpc had significantly lower (*p* < .05) respiratory sign scores even compared with the Vac2B/Ch1‐2 group. Pigs in the Vac1A/Ch1‐2 group had significantly lower (*p* < .05) scores compared to the UnVac/Ch1‐2 group even at 12 dpc. Pigs in the UnVac/UnCh group maintained normal temperatures without respiratory disease symptom throughout the study (Figure 2b). In summary, pigs vaccinated with either PRRSV‐1 MLV vaccine or PRRSV‐2 MLV vaccines had lower rectal temperatures and better respiratory clinical scores compared to unvaccinated challenged pigs.

### 3.2 Quantification of PRRSV RNA

Quantitative RT‐PCR was used to determine the number of PRRSV genomic copies in serum samples of pigs from all groups. Genomic copies of the vaccine virus were detected in the sera of all the vaccinated pigs from the Vac1A/Ch1‐2, Vac1B/Ch1‐2, Vac2A/Ch1‐2 and Vac2B/Ch1‐2 groups at −21 dpc (14 days post vaccination). No cross‐contamination of any of the vaccine viruses in any of the vaccinated groups was observed. No vaccine virus was detected in the serum or oral fluids of pigs from the Vac1A/Ch1‐2, Vac1B/Ch1‐2, Vac2A/Ch1‐2 and Vac2B/Ch1‐2 groups at 0 dpc (35 days post vaccination). No vaccine virus was detected in the serum and oral fluids of pigs in the UnVac/Ch1‐2 and UnVac/UnCh groups at −21 and 0 dpc.

**Figure 3:**
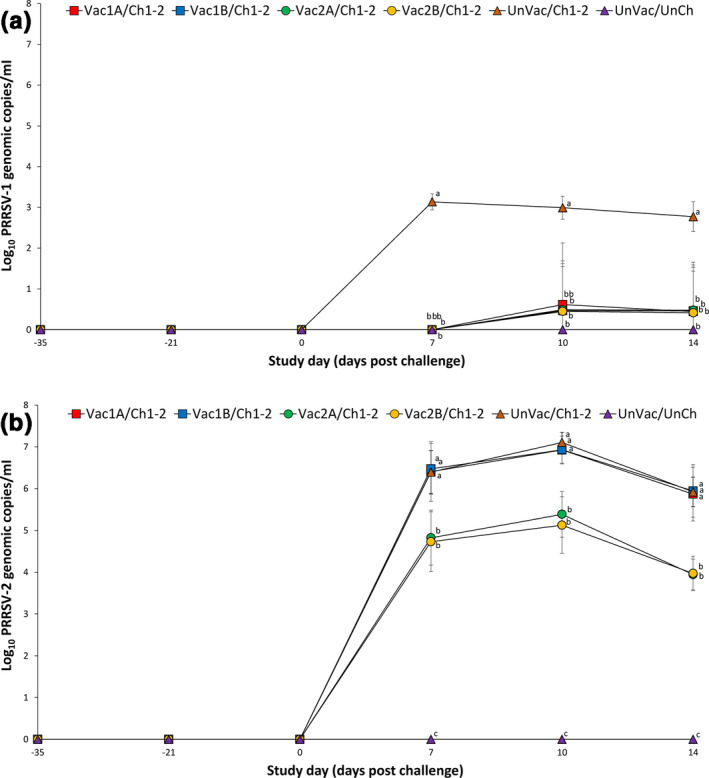
Mean (with standard deviation) of the number of genomic copies of PRRSV‐1 RNA (a) and PRRSV‐2 RNA (b) in serum samples of pigs from six groups. Different letters (a, b and c) at days post challenge indicate significant differences among groups

Pigs from the Vac1A/Ch1‐2, Vac1B/Ch1‐2, Vac2A/Ch1‐2 and Vac2B/Ch1‐2 groups had significantly fewer (*p* < .05) genomic copies of PRRSV‐1 in their sera compared to the UnVac/Ch1‐2 group at 7, 10 and 14 dpc (Figure 3a). Pigs from the Vac2A/Ch1‐2 and Vac2B/Ch1‐2 groups had significantly fewer (*p* < .05) genomic copies of PRRSV‐2 in their sera compared to the Vac1A/Ch1‐2, Vac1B/Ch1‐2 and UnVac/Ch1‐2 groups at 7, 10 and 14 dpc. No PRRSV genomes of any type were detected in the sera of pigs from the UnVac/UnCh group throughout the study (Figure 3b). In summary, pigs vaccinated with the PRRSV‐1 MLV vaccines reduced PRRSV‐1 viraemia only while pigs vaccinated with the PRRSV‐2 MLV vaccines reduced PRRSV‐1 and PRRSV‐2 viraemia in dually challenged pigs.

### 3.3 Serology

**Figure 4:**
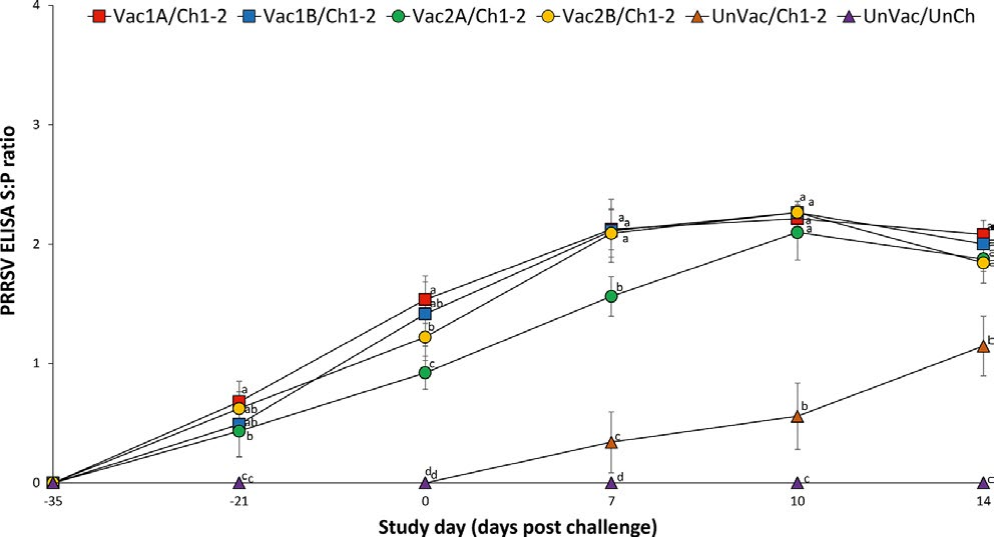
Mean (with standard deviation) for PRRSV ELISA sample‐to‐positive (S/P) ratio in serum samples of pig from six groups. Different letters (a, b and c) at days post challenge indicate significant differences among groups

At the time of PRRSV vaccination (study day –35), pigs from all six groups were confirmed seronegative with a PRRSV ELISA. Anti‐PRRSV antibodies were detected in vaccinated pigs only before challenge. Pigs from the Vac1A/Ch1‐2, Vac1B/Ch1‐2, Vac2A/Ch1‐2 and Vac2B/Ch1‐2 groups had significantly higher (*p* < .05) PRRSV ELISA S/P ratio at –21, 0, 7, 10 and 14 dpc compared to the UnVac/Ch1‐2 group. Pigs from the Vac1A/Ch1‐2 group had significantly higher (*p* < .05) PRRSV ELISA S/P ratio at –21 dpc compared to the Vac2A/Ch1‐2 group. Pigs from Vac1A/Ch1‐2, Vac1B/Ch1‐2 and Vac2B/Ch1‐2 groups had significantly higher (*p* < .05) PRRSV ELISA S/P ratio at 0 and 7 dpc compared to the Vac2A/Ch1‐2 group. Pigs from the Vac1A/Ch1‐2 group had significantly higher (*p* < .05) PRRSV ELISA S/P ratio at 0 dpc compared to the Vac2B/Ch1‐2 group. Anti‐PRRSV antibodies were not detected in pigs from the UnVac/UnCh group at any time (Figure 4). In summary, pigs vaccinated with either PRRSV‐1 MLV vaccine or PRRSV‐2 MLV vaccines induced higher levels of anti‐PRRSV antibodies compared to unvaccinated dually challenged pigs.

### 3.4 Interferon‐γ secreting cells

**Figure 5:**
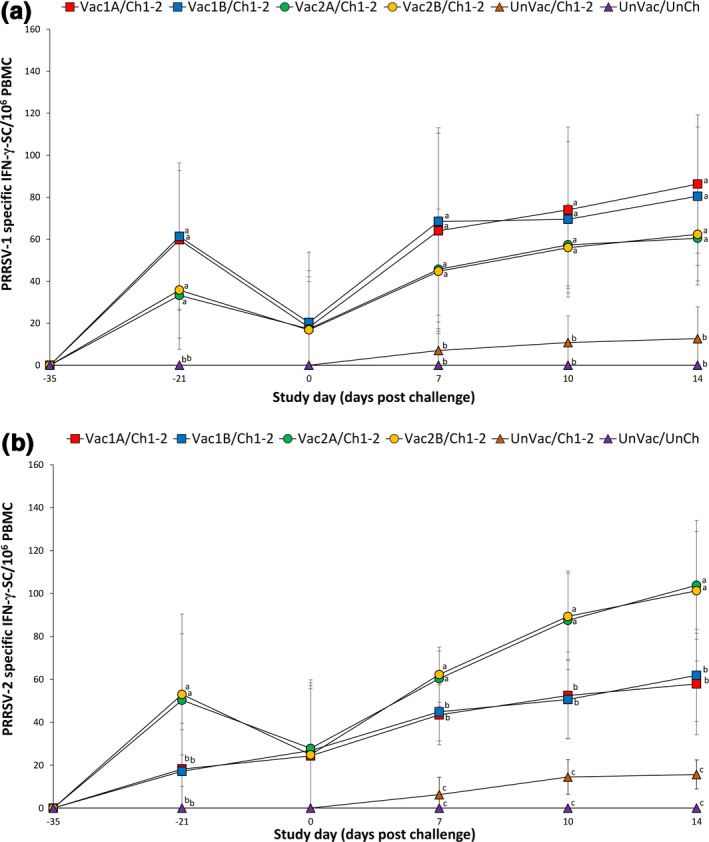
Mean (with standard deviation) of PRRSV‐1 specific IFN‐γ‐SC (a) and PRRSV‐2 specific IFN‐γ‐SC (b) in peripheral blood mononuclear cells of pigs from six groups. Different letters (a, b and c) at days post challenge indicate significant differences among groups

To evaluate activation of T cells after vaccination, the number of IFN‐γ‐SCs was compared. Pigs from the Vac1A/Ch1‐2, Vac1B/Ch1‐2, Vac2A/Ch1‐2 and Vac2B/Ch1‐2 groups produced a significantly higher (*p* < .05) number of PRRSV‐1 specific IFN‐γ‐SC compared to the UnVac/Ch1‐2 group at −21, 7, 10 and 14 dpc (Figure 5a). Pigs from the Vac2A/Ch1‐2 and Vac2B/Ch1‐2 groups produced a significantly higher (*p* < .05) number of PRRSV‐2 specific IFN‐γ‐SC compared to the Vac1A/Ch1‐2, Vac1B/Ch1‐2, and UnVac/Ch1‐2 group at −21, 7, 10 and 14 dpc. Pigs from the Vac1A/Ch1‐2 and Vac1B/Ch1‐2 groups produced a significantly higher (*p* < .05) number of PRRSV‐2 specific IFN‐γ‐SC compared to the UnVac/Ch1‐2 group at 7, 10 and 14 dpc. The mean numbers of PRRSV‐1 and PRRSV‐2 specific IFN‐γ‐SC remained at basal levels (< 20 cells/10^6^ PBMC) in all pigs from the UnVac/UnCh group throughout the study (Figure 5b). In summary, vaccination of pigs with either PRRSV‐1 MLV vaccines or PRRSV‐2 MLV vaccines elicited equal numbers of PRRSV‐1 specific IFN‐γ‐SC while vaccination of pigs with PRRSV‐2 MLV vaccines elicited higher frequency of PRRSV‐2 specific IFN‐γ‐SC compared to vaccination of pigs with PRRSV‐1 MLV vaccines.

### 3.5 Pathology

Macroscopic and microscopic lung lesions were also evaluated at 7 and 14 dpc. Pigs from the Vac2A/Ch1‐2 and Vac2B/Ch1‐2 groups showed significantly lower (*p* < .05) mean macroscopic and microscopic lung lesion scores at 7 and 14 dpc compared to pigs from the Vac1A/Ch1‐2, Vac1B/Ch1‐2 and UnVac/Ch1‐2 groups. No lung lesions were observed in any of the pigs from the UnVac/UnCh group (Table 1). In summary, pigs vaccinated with PRRSV‐2 MLV vaccines reduced macroscopic and microscopic lung lesions compared to vaccination of pigs with PRRSV‐1 MLV vaccines.

## 4 DISCUSSION

In this study, we compared the efficacy of four commercial vaccines against respiratory disease in growing pigs after heterologous dual challenge. All four vaccines are MLVs and two are based on PRRSV‐1 and two on PRRSV‐2. Pigs vaccinated with the PRRSV‐2 MLV vaccines had lower rectal temperatures, lower viraemia and better respiratory disease scores with reduced lung lesions compared to the control group, suggesting a partial protection against dual challenge with similar efficacies. The PRRSV‐1 MLV vaccines appeared to be ineffective against heterologous dual challenge. Care must be taken to interpret the results of lower rectal temperatures in vaccinated pigs. The differences in body temperature may have no significant meaning if the temperature was not significantly above the fever threshold.

It is important to note that according to a previous study, the same PRRSV‐2 challenge strain used here caused gross and microscopic lung lesions comparable to dual challenge (Choi et al., 2015). PRRSV‐1 viraemia was also similarly reduced in the dual challenge compared to PRRSV‐1 infection alone after vaccination (Choi et al., 2015). Therefore, we believe that the PRRSV‐1 strain used in the current study does not contribute to respiratory disease severity in a dual infection. Our preliminary data using combinations of three other strains of each PRRSV‐1 and PRRSV‐2 showed similar results (data not shown). This again suggests that PRRSV‐2 may play a more prominent role than PRRSV‐1 in respiratory disease and pathological lung lesions from a dual infection with Korean PRRSV‐1 and PRRSV‐2 strains. For this reason, the effect of PRRSV‐2 vaccine on PRRSV‐1 virus may be inconclusive, as the PRRSV‐2 virus was dominate over the PRRSV‐1 virus in dually infected pigs. In the previous single challenge study, the same PRRSV‐2 MLV vaccines used in this study can protect against both PRRSV‐1 and PRRSV‐2 challenge (Oh et al., 2019). Therefore, the PRRSV‐2 MLV appeared to be effective against heterologous dual challenge.

There is the possibility for PRRSV MLV‐vaccinated pigs to shed and thus disseminate vaccine virus among pig populations. To avoid the potential cross‐contamination of vaccine viruses from the four different PRRSV MLV vaccines, each vaccinated group (Vac1A/Ch1‐2, Vac1B/Ch1‐2, Vac2A/Ch1‐2 and Vac2B/Ch1‐2) was housed in separate rooms until dual challenge of PRRSV‐1 and PRRSV‐2. After dual challenge, all challenged groups (Vac1A/Ch1‐2, Vac1B/Ch1‐2, Vac2A/Ch1‐2, Vac2B/Ch1‐2 and UnVac/Ch1‐2) were co‐mingled. No vaccine virus could be detected in serum and oral fluids in any of the four vaccinated groups at the time of dual challenge with PRRSV‐1 and PRRSV‐2, 35 days post vaccination. These results are consistent with a previous study, where no vaccine virus, from the same four MLV vaccines used here, could be detected in the serum of the vaccinated pigs at 28 days post vaccination (Díaz & Mateu, 2015). Altogether, this suggests that the PRRSV detected in the serum of the vaccinated pigs after the dual challenge is the challenge strains.

Neutralizing antibodies and T‐cell responses typically play an important role in reducing PRRSV viraemia and controlling PRRSV infection (Madapong et al., 2017). However, there is recent evidence from studies with single PRRSV‐1 or PRRSV‐2 infection that PRRSV viraemia is often reduced even before neutralizing antibodies are detected in infected and vaccinated pigs (Madapong et al., 2017; Mateu & Diaz, 2008; Mengeling, Lager, Vorwald, & Clouser, 2003; Nelson, Christopher‐Hennings, & Benfield, 1994). Vaccination with all four vaccines used in this study did not generate any detectable neutralizing antibodies until at least 14 days post single challenge with either PRRSV‐1 or PRRSV‐2 (Jeong, Choi, Kang, Park, & Chae, 2016; Kim et al., 2015; Park et al., 2014). Taken together, this suggests that neutralizing antibodies are not essential for PRRSV clearance (Kimman, Cornelissen, Moormann, Rebel, & Stockhofe‐Zurwieden, 2009; Mateu & Diaz, 2008). In contrast, T‐cell responses as measured by an increase in the number of IFN‐γ‐SC directly correlates with the reduction of PRRSV viraemia (Correas, Osorio, Steffen, Pattnaik, & Vu, 2017; Meier et al., 2003). In the present study, vaccination of pigs with either of the two PRRSV‐1 MLV vaccines or the two PRRSV‐2 MLV vaccines elicited equal numbers of PRRSV‐1 specific IFN‐γ‐SC. In contrast, vaccination of pigs with either of the two PRRSV‐2 MLV vaccines elicited higher frequency of PRRSV‐2 specific IFN‐γ‐SC compared to vaccination of pigs with either of the two PRRSV‐1 MLV vaccines. These data can explain why PRRSV‐1 MLV vaccines are able to reduce PRRSV‐1 viraemia only while PRRSV‐2 MLV vaccines are able to reduce both PRRSV‐1 and PRRSV‐2 viraemia.

To date, in Korea, there are four PRRSV MLV vaccines that have been licensed for commercial use. Two are based on PRRSV‐1 and two on PRRSV‐2. Therefore, it was important to compare the efficacy of all four under the same experimental conditions. Control of both species of PRRSV by a single vaccine is the number one goal for swine producers because the number of pigs that are co‐infected with both species is rapidly increasing. Different levels of cross protection between PRRSV‐1 and PRRSV‐2 MLV vaccines can provide swine practitioners and producers with significant clinical information on how to select the proper vaccines to protect their livestock against respiratory disease caused by co‐infection with both species of PRRSV.

## CONFLICT OF INTEREST

The authors declare no conflict of interests with respect to their authorship or the publication of this article.

## AUTHOR CONTRIBUTION

**Taehwan Oh:** Conceptualization; Data curation; Investigation; Methodology; Writing‐original draft. **Su‐Jin Park:** Conceptualization; Formal analysis; Investigation; Software; Writing‐original draft. **Hyejean Cho:** Data curation; Resources; Software; Visualization. **Siyeon Yang:** Methodology; Software. **Hee Jin Ham:** Formal analysis; Validation. **C. Chae:** Conceptualization; Project administration; Supervision; Writing‐review & editing.

## Ethical Statement

All of the methods were previously approved by the Seoul National University Institutional Animal Care and Use, and Ethics Committee. Sample collection was carried out according to the animal welfare code of Korea.

## Data Availability

The data that support the findings of this study are available from the corresponding author upon reasonable request.
